# Mini-implants and the efficiency of Herbst treatment: a preliminary study

**DOI:** 10.1186/2196-1042-14-21

**Published:** 2013-07-31

**Authors:** Cesare Luzi, Valeriano Luzi, Birte Melsen

**Affiliations:** Via Savoia 35/a, Rome, 00198 Italy; Dental Institute, University of Rome La Sapienza, Viale Regina Elena 287/A, Rome, 00161 Italy; Department of Orthodontics, School of Dentistry, University of Aarhus, Vennelyst Boulevard 9, Aarhus, C 8000 Denmark

## Abstract

**Background:**

The purpose of this study is to present the use of a modified Herbst appliance in association with temporary anchorage devices (TADs) in order to enhance the correction of skeletal class II malocclusions.

**Methods:**

Ten consecutive adolescents scheduled for Herbst treatment were assigned to two treatment groups. Five cases were treated with a modified miniscrew-supported Herbst appliance (experimental group (EG)) and five cases with a conventional cast Herbst appliance (control group (CG)). In all cases, the Herbst was kept in place for 9 months and was followed by fixed appliances until class I relationships were achieved. The initial (T1) and final (T2) lower incisor inclination on lateral headfilms were analyzed for each case, and the mean increase for the five EG patients and the five CG patients were compared.

**Results:**

The mean increase in lower incisor inclination at the end of treatment was 1° (range 0° to 2°) for the EG and 7° (range 4° to 10°) for the CG.

**Conclusions:**

The rational association of TADs with the Herbst appliance can optimize treatment efficiency and skeletal response by reducing the occurrence of excessive lower incisor proclination.

## Background

The introduction of noncompliance systems has markedly influenced class II treatment [1]. Devices with the purpose of distalizing upper molars [2–7] as well as devices with the purpose of advancing the mandible [8–13] have become very popular in the last two decades. However, in spite of the popularity of these devices, they all lead to some anchorage loss and undesirable side effects as the anchorage teeth do never exhibit complete stability. Herbst treatment, in particular, generally features an unwanted proclination of the lower anterior segment [14–16]. The introduction of temporary anchorage devices (TADs) has recently brought the option of absolute anchorage control to daily clinical practice. While this benefit has been extended to distalizing devices [17–20], eliminating anterior anchorage loss, and generating new and more efficient treatment protocols, the association of bite-jumping devices and TADs has been discussed [21] but not yet evaluated in a controlled clinical study. The aim of this study, therefore, is to evaluate whether the association of a modified Herbst appliance and TADs can enhance the correction of skeletal class II malocclusion, avoiding the undesirable proclination of the lower anterior teeth.

## Methods

**Figure 1:**
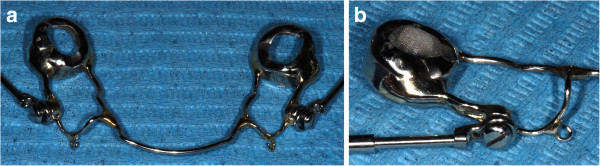
**The lower cast structure of the modified Herbst with the bilateral hooks (a, b).**

**Figure 2:**
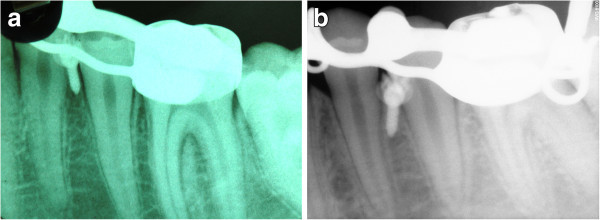
**Mini-implants inserted between the lower first and second premolars.** They were inserted in patients RR **(a)** and NP **(b)** from the EG.

**Figure 3:**
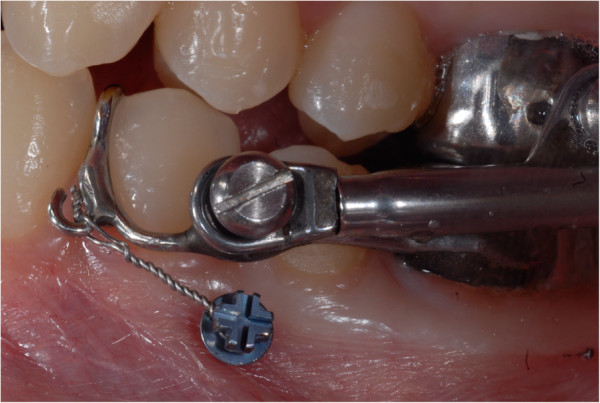
**The modified Herbst connected to the TAD with the 0.12-mm stainless steel ligature.**

Ten consecutive adolescents (age 11 to 15 years) scheduled for Herbst treatment in a private practice in Rome, Italy were assigned to two treatment groups. All patients presented at treatment start a class II division 1 malocclusion with increased overjet (range 5 to 12 mm). Patients with mild/severe crowding of the lower arch or requiring extractions were excluded from the sample. Five cases were treated with a modified mini-implant-supported Herbst (experimental group (EG)) and five cases with a conventional cast Herbst (control group (CG)) by the same clinician (CL). The assignment to the groups was based on the initial degree of lower incisor inclination with the five initial, most severe inclinations assigned to the EG (Table 1). The modified Herbst of the EG was constructed so that a bilateral customized hook was soldered on the cast structure facilitating the connection to the TADs (Figure 1). The presence of the hooks was the only difference from the Herbst of the CG. Following the insertion of the Herbst, in the EG, two miniscrews (thread length 6.0 mm, diameter 1.5 mm) were inserted under local anesthesia on the lower buccal cortex either between the roots of the first and second premolars bilaterally or between the roots of the second premolars and first molars bilaterally (Figure 2) and were tightly connected to the customized hooks on the Herbst appliance with a 0.12-mm stainless steel ligature (Figure 3). Chlorhexidine rinse was prescribed for 1 week daily after tooth brushing. In all cases, the Herbst was kept in place for 9 months and followed by fixed appliances (total treatment time 19 to 28 months) with the same Roth-type bracket prescription. During the Herbst phase, the appliance was activated stepwise, 2 mm every 8 weeks until incisors reached an edge-to-edge position. At the end of treatment, the initial (T1) and final (T2) inclination of the lower incisors (L1/Go-GN) were measured on the lateral headfilms by the same experienced operator (CL), and the changes occurring in the five EG patients and the five CG patients were compared. All measurements were performed blinded: the 20 cephalograms were mixed and then singularly measured twice without the operator knowing whose headfilm was under measurement at that moment.

**Table 1:** **Values of initial and final lower incisor inclinations and ANB angles of all the patients**

Group	Patient	Initial L1/Go-GN	Final L1/Go-GN	Initial ANB angle	Final ANB angle
EG	NP	109°	109°	9°	4°
	FM	107°	109°	8°	5°
	RR	102°	102°	5°	3°
	TM	105°	107°	9°	4°
	MS	112°	113°	7°	4°
CG	SC	97°	104°	7°	4°
	MED	99°	103°	6°	4°
	MR	101°	111°	9°	7°
	JG	96°	105°	6°	5°
	GD	90°	95°	7°	5°

A point, nasion, B point = ANB angle.

## Results and discussion

### Results

**Figure 4:**
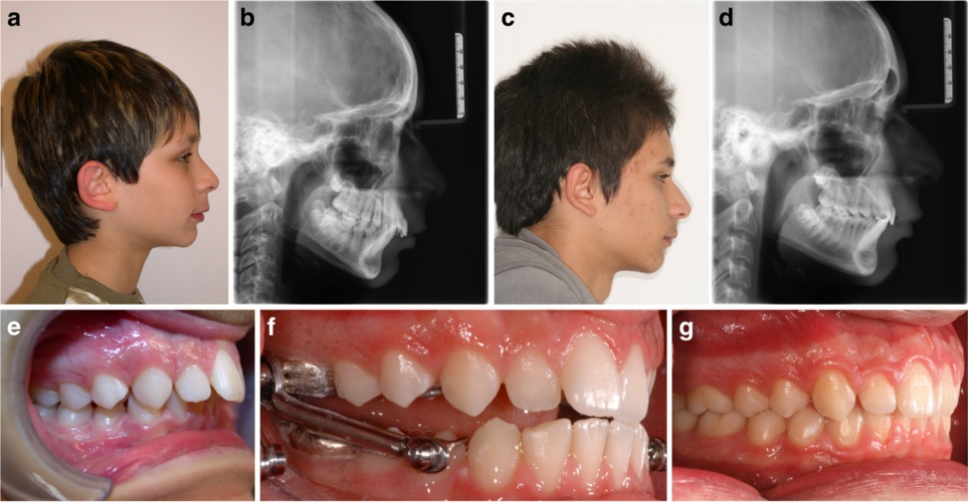
**Patient JG from the CG.** Pretreatment profile **(a)** and cephalogram **(b)**. Posttreatment profile **(c)** and cephalogram **(d)**. Lateral intraoral views pretreatment **(e)**, with the Herbst **(f)**, and posttreatment **(g)**. The overjet is reduced mainly by dentoalveolar compensations.

**Figure 5:**
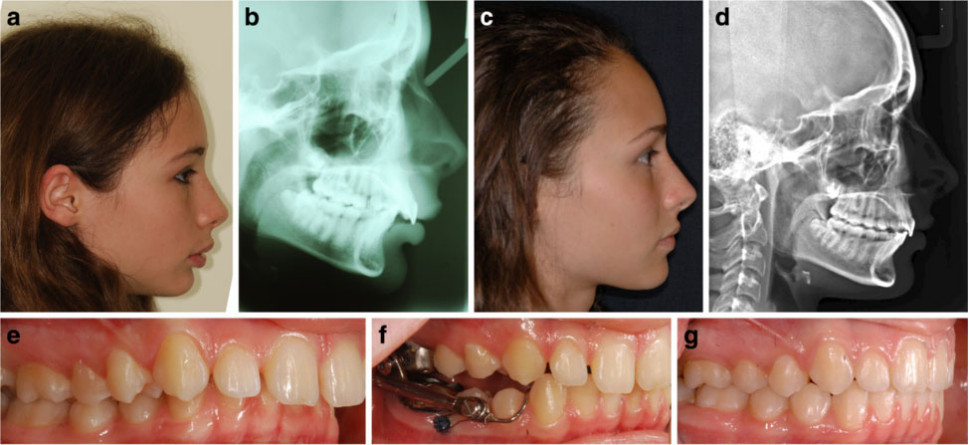
**Patient FM from the EG.** Pretreatment profile **(a)** and cephalogram **(b)**. Posttreatment profile **(c)** and cephalogram **(d)**. Lateral intraoral views pretreatment **(e)**, with the Herbst connected to TADs **(f)**, and post-treatment **(g)**. The pretreatment increased lower incisor inclination and was controlled during treatment; the overjet was reduced with maximum skeletal response [22].

Two miniscrews in two different patients of the EG lost stability during treatment. One was immediately substituted with a new one inserted in an adjacent site, while the second one was tightened deeper until stability was achieved again. All patients achieved both skeletal improvement and dental correction to class I relationships (Figures 4 and 5). The mean increase in lower incisor inclination at the end of treatment was 1° (range 0° to 2°) in the EG and 7° (range 4° to 10°) in the CG. The mean decrease in the A point, nasion, B point (ANB) angle at the end of treatment was 3.6° (range 2° to 5°) in the EG and 2° (range 1° to 3°) in the CG (Table 1). As the sample size was very small, no statistical analysis was performed.

### Discussion

This preliminary study describes the association of a modified Herbst appliance and TADs comparing the effects of this new protocol to traditional Herbst treatment. To date, with a history of over 100 years, the Herbst appliance is still considered a state-of-the-art solution and the most used noncompliance device for class II treatment [23–26]. All fixed bite-jumping devices generate both skeletal and dental effects, resulting as a main side effect in a proclination of the lower incisors, contributing to overjet reduction [14–16]. This undesired effect can minimize the skeletal effects and impede the desired forward displacement of the mandible, thereby preventing the establishment of a solid class I final dental relationship and a tight intercuspation. Although many attempts have been made to avoid lower incisor proclination, e.g., with the use of cast mandibular appliances, archwires with torque bends, or brackets with selective torques, absolute anchorage control has not yet been achieved and loss of mandibular anchorage can be anticipated [27, 28]. This is especially undesirable in patients where the lower incisors already exhibit a compensatory proclination at the start of treatment, for which reason, further proclination should be avoided during the correction of the sagittal discrepancy. This was taken into consideration when the patients were assigned to the experimental or the control group. A randomized assignment to groups was considered unethical, and the five cases with the greatest proclination of the lower incisors measured to the mandibular plane were therefore assigned to the EG. To reduce bias, the same bracket prescription was used in all the cases, and patients either requiring extractions or with lower arch crowding greater than 4 mm were excluded from the sample. Therefore, the changes in lower incisor position that resulted from leveling and alignment or the space closure phases were excluded. Most cephalometric errors are either due to experience of the operator or to landmark identification [29]. All landmark identification and measurements were carried out twice by the same experienced operator. In case of discrepancy between the first and second measurements, a third measurement was then performed.

Although TADs have become a daily clinical tool in orthodontics, their use, in combination with the Herbst appliance, has not yet been standardized into a treatment protocol or reported in clinical trials. This preliminary study describes how a combination of miniscrews and Herbst appliance can control the proclination of the lower incisors during treatment. The mandibular alveolar process has optimal cortical bone thickness and adequate inter-root distance for insertion of miniscrews with standard characteristics (1.5-mm diameter and 6-mm thread length) [30, 31]. Following the insertion and in the presence of primary stability, a stiff connection to the Herbst appliance was required. This was achieved by means of a 0.12-mm stainless steel ligature wire. In this way, the Herbst was directly connected to the lower dentition and indirectly to the lower basal bone with the aim of enhancing the skeletal effect and reducing the dentoalveolar compensations in the mandibular arch. Direct orthopedic load on miniscrews should be avoided as it most likely would increase the risk of failure of these devices designed to withstand standard orthodontic forces [32]. In all cases, the Herbst appliance was kept in place for a minimum of 9 months (mean duration of Herbst phase 9.6 months) to induce adequate skeletal and neuromuscular adaptation [33]. The stability of the Herbst in both groups and the stability of the TADs and tightness of the ligature wires in the EG were routinely checked monthly. The two miniscrews that lost stability were either positioned too close to a dental root [34] or loosened due to adverse local conditions (poor bone quality, inflammation, etc.). It is well known that mini-implants experience failure, and various success rates are reported without significant differences between immediately loaded or delayed loaded samples [35]. The conditions of the load (immediate and indirect) could not be blamed for the loss of stability. The success rates of the insertion sites, between the mandibular premolars and the second premolar and first molar, have not shown to differ from other mandibular or maxillary sites in growing patients [36]. Patient gender and side of insertion can also be considered irrelevant in relationship to success rates. Although the mandibular inter-radicular space between the first and second premolars is, on average, greater than the one between the second premolar and first molar [37], individual variations should be considered and periapical radiographs should always be used for implant site determination.

At the end of treatment, all cases displayed class I canine and molar relationships and reduction of the ANB angle. The results thus confirm the efficiency of the Herbst appliance in the correction of class II malocclusions [38]. This corroborates the findings of Schaefer et al. [39] who reported normalization of the dentoskeletal parameters following overall treatment in their stainless steel crown Herbst sample. However, there was a clear difference of the change in lower incisor inclination between the two groups studied. The side effects were reduced in the EG (Table 1). While the mean increase in the inclination of the lower incisors from T1 to T2 in the CG is comparable to the one reported in other similar studies with greater sample size [14, 39], as a result of the absolute anchorage delivered by the TADs, this proclination was smaller in the EG. In addition, the mean reduction in ANB angle was greater in the EG (Table 1), confirming the fact that reducing the dentoalveolar compensations during Herbst treatment can help to achieve a greater skeletal effect. Although the EG showed reduced dentoalveolar compensations compared to the CG, the results should be interpreted with caution due to the small sample size.

## Conclusions

The association of TADs with the Herbst appliance can be applied in order to reduce undesirable proclination of lower incisors and enhance the skeletal response. The simple use of mini-implants makes it possible for the orthodontist to develop a new ideal treatment protocol facilitating the correction of class II malocclusions in those adolescents requiring mandibular advancement with minimal dentoalveolar compensations. Further studies and clinical trials with a larger sample size are recommended to confirm the results of this preliminary study.

## Consent

Written informed consent was obtained from the patients for publication of this report and any accompanying images.
